# Predictive value of type D personality for cardiac events in Chinese patients with acute myocardial infarction

**DOI:** 10.1186/s12872-023-03598-w

**Published:** 2023-11-14

**Authors:** Jiahui Li, Wenjing Wu, Nan Li, Jian Wang, Liyuan Zu, Xiaojun Ye

**Affiliations:** Department of Cardiology, China-Japan Friendship Hospital, 2 Yinghuayuan Street East, Chaoyang District, 100029 Beijing, China

**Keywords:** Type D personality, Myocardial Infarction, Chinese

## Abstract

**Background:**

Our study aimed to investigate the association between type D personality and adverse cardiac events in chinese patients after acute myocardial infarction (AMI).

**Methods:**

Patients with AMI admitted to cardiac care unit (CCU) of China-Japan Friendship Hospital, Beijing, China between January 2016 and December 2017 were enrolled. 257 patients completed psychological questionnaires at enrollment. Type D personality was assessed with 14-item Type D Scale-14 (DS14). Anxiety and depression were quantified using Hospital Anxiety and Depression Scale (HADS). Multivariable logistic regression analysis was used to determine the independent predictors of in-hospital major adverse cardiac events (MACEs), while cox regression analysis was used to evaluate post-discharge endpoints.

**Results:**

54 patients (21%) were classified as Type D personality defined by the combination of a negative affectivity (NA) score ≥ 10 and a social inhibition (SI) score ≥ 10 on the DS14. Patients with Type D personality displayed significantly higher scores of anxiety (7.4 ± 3.1 vs. 4.2 ± 3.1, p < .001) and depression (7.2 ± 3.8 vs. 4.0 ± 3.4, p < .001). AMI patients with Type D personality had higher prevalence rates of anxiety (χ2 = 30.095, P < .001) and depression (χ2 = 27.082, P < .001). Type D group also displayed a significantly higher level of blood lipoprotein(a) (177.2 ± 200.7 vs. 118.1 ± 255.7 mg/L, P = .048). The incidence of in-hospital MACEs was higher in type D than in non-Type D patients (24.1% vs. 11.3%, χ2 = 5.751, P = .026). Multivariable logistic regression showed three significant independent predictors of in-hospital MACEs: age [odds ratio(OR) = 1.055; 95%CI 1.016–1.095, p = .004], type-D personality(OR 3.332; 95% CI 1.149–9.661, p = .014) and killip classification(OR 2.275, 95% CI 1.506–3.437, p < .001). The average follow-up time was 31 (23-37.5) months. Type D patients had higher incidences of post-discharge events(23.1% vs. 11.5%, p = .032). In the analysis of post-discharge events by Cox regression, χ2 of the Cox regression equation was 16.795 (P = .032). Smoking (HR 2.602; 95% CI1.266–5.347, p = .009) and type-D personality (HR 2.265; 95%CI 1.028–4.988, p = .042) were independent predictors of long-term cardiac events. Kaplan–Meier curves showed significant difference in event-free survival between type D and non-type D group (p = .043).

**Conclusions:**

Type D personality is an independent predictor of in-hospital and post-discharge cardiac events after AMI in Chinese patients.

**Supplementary Information:**

The online version contains supplementary material available at 10.1186/s12872-023-03598-w.

## Background

Despite great advances in the diagnosis and therapy of coronary heart disease (CHD), patients still suffer from adverse cardiac events. Psychosocial risk factors are equally important in risk prediction for CHD compared with sex, metabolic and behavioural risk factors [1]. Type D (“distressed”) personality is characterized by negative affectivity (NA) and social inhibition (SI). NA refers to the tendency of experiencing negative thoughts, feelings and emotions, while SI people tend to feel inhibited and insecure with difficulty in expressing these emotions in social situations [2]. Type D personality is associated with more severe coronary artery calcification [3], impaired endothelial function [4], hyperlipidemia [5], unhealthy lifestyles [6] and greater risk for cardiac events in CHD [7]. A recent individual patient-data meta-analysis combined the data of 19 previously published prospective cohort studies and proved that Type D personality is related to adverse events in CHD [8]. Acute myocardial infarcrtion (AMI) is the most severe type of CHD and the incidence of it is still increasing. In the US, during the period from 2001 to 2011, in-hospital mortality after AMI did not change for patients who received percutanous coronary intervention (PCI) [9]. The characteristics of type D personality have been found to have a statistically significant association with AMI [10]. However, the predictive value of type D personality remains controversial and inconclusive. Large heterogeneity exists between type D studies and negative findings have been reported [11]. Results varied depending on the selected population, age, ethnicity, choice of endpoints and methods to assess type D personality [12]. Data about the impact of type D personality on AMI in chinese patients is also limited.The aim of our study was to evaluate the predictive value of type D personality for cardiac events in chinese patients with AMI.

## Methods

### Study design and paticipants

Patients with AMI admitted to cardiac care unit (CCU) of China-Japan Friendship Hospital, Beijing, China between January 2016 and December 2017 were enrolled. A majority of the patients experienced both an AMI and subsequently underwent revascularization. Patients with cancer or other life-threatening medical conditions were excluded. At baseline, 257 patients provided written informed consent and completed psychological questionnaires at enrollment by theirselves or with the nurses’ help. This study was approved by the Ethics Committee of China-Japan Friendship Hospital in Peking of China and performed in accordance with the principles of the Declaration of Helsinki revised in 2013. The original data will be shared on reasonable request by contacting the corresponding author.

### Type D personality assessment

Type D Personality was assessed at baseline with the Chinese version of 14-item Type D Scale-14 (DS14), which contains 7-item NA and SI subscales [2, 13]. Items are rated on a 5-point scale ranging from 0 = false to 4 = true. A cut-off ≥ 10 on the NA and SI measures identifies individuals with elevated trait levels, and individuals with a score ≥ 10 on both scales are categorized as type D. The DS-14 is a valid measure of NA and SI in Chinese population [13]. In our study, NA and SI subscales are internally consistent with the Cronbach’s alpha 0.82 and 0.80 respectively.

### Hospital anxiety and depression measures

Assessment of psychological status was quantified using Hospital Anxiety and Depression Scale (HADS), which is composed of 14 items and contains two subscales: anxiety (HADS-A) and depression (HADS-D) [13, 14]. Each item is from 0 (no symptoms) to 3 (maximum symptom level). The maximum score for each subscale is 21 and scores 0–7 on each subscale are considered normal. A cutoff score ≥ 8 was used for both subscales to identify patients with likely anxiety and depression. HADS-A and HADS-D are internally consistent with the Cronbach’s alpha 0.78 and 0.79 respectively.

### In-hospital cardiac events and post-discharge endpoints

Most literatures recognized that ventricular tachycardia / fibrillation, acute recurrent myocardial ischemia, reinfarction, cardiogenic shock, acute pulmonary edema and cardiac death are the main in-hospital complications of AMI [15]. So in-hospital events were major adverse cardiac events (MACEs; a composite of ventricular tachycardia/fibrillation, acute recurrent myocardial ischemia, reinfarction, cardiogenic shock, acute pulmonary edema and cardiac death). The follow-up interval was fixed at 2-3years. Patients and their families were contacted by telephone to determine the endpoints. Information on mortality, nonfatal myocardial infarction (MI), PCI and CABG were extracted from hospital records and the patient’s attending physician was involved to determine the cause of death. The post-discharge endpoints were defined as a composite of unstable angina, reinfarction, cardiac revascularization (PCI/CABG) and cardiac death [16].

### Statistical analysis

All statistical analyses were performed using SPSS version 22.0 software (SPSS Inc., Chicago, IL, USA). Numerical variables were expressed as mean ± standard deviation when normally distributed and Student’s t test was used for comparison between two groups. When not normally distributed, data were expressed as median ± interquartile range (IQR) and Mann-Whitney U test was used. Frequencies and percentages were used to express categorical variables, which were analyzed by Chi square test. Taking the in-hospital MACEs as the outcome variable, and multivariable logistic regression analysis was used to calculate odds ratio (OR) and determine the independent predictors of in-hospital MACEs. We included all baseline variables and made a stepwise selection. The model included variables (age, left ventricular ejection fraction, killip class, type-D persionality, HADS-A, HADS-D, No. of diseased vessels, fasting blood glucose, low-density lipoprotein cholesterol). HADS was modeled to determine the independent predictors of in-hospital MACEs, which was not used to evaluate post-discharge endpoints because anxiety and depression states are dynamically changing and it is believed that the in-hospital score can not have a significant impact on the long-term prognosis. We used separate scores for anxiety and depression. Each subscale was a separate continuous variable. The Hosmer Limeshow Goodness of fit test was selected, and the chi-square test of regression equation model fitting showed that the P-value was less than 0.05, indicating a good fitting of the regression model. Cox regression analysis was used to evaluate post-discharge endpoints. P < .05 was considered significant.

## Results

### Baseline characteristics

**Fig. 1:**
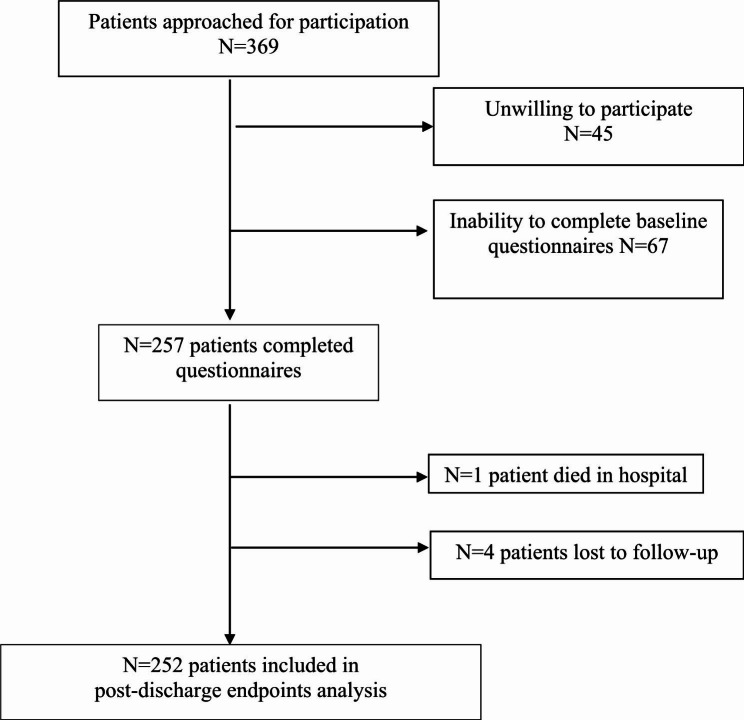
Flow chart of patient screening

**Fig. 2:**
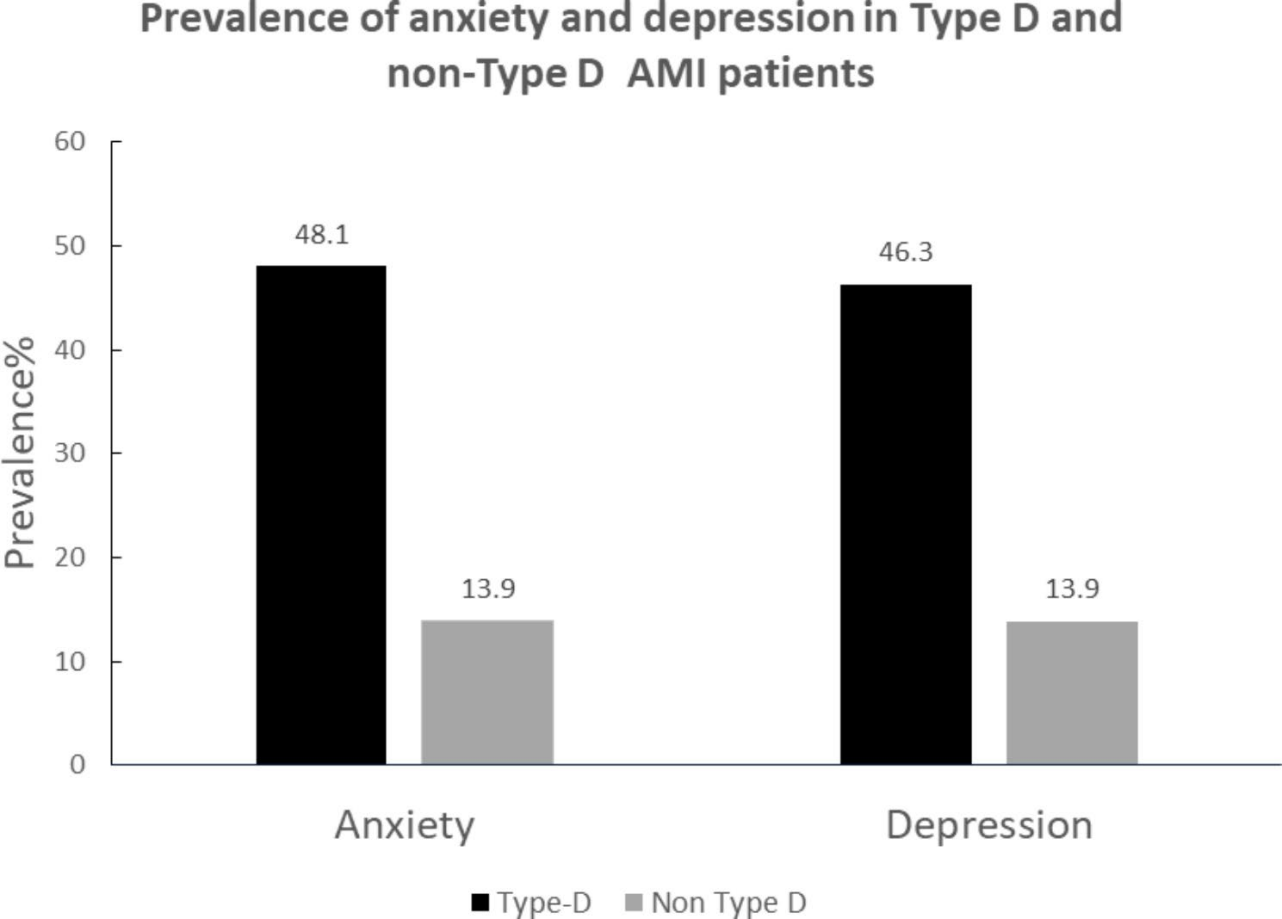
Prevalence of anxiety and depression in Type D and non-Type D patients

Flow chart of patient screening was shown in Fig. 1. Table 1 presented the characteristics of the 257 patients included in this study. The mean age was 64.3 ± 13.7years, 75% were men, and a majority of patients underwent PCI or CABG. 54 patients (21.0%) were classified as type D personality defined by the combination of a NA score ≥ 10 and a SI score ≥ 10 on the DS14, all other patients were classified as non-type D with 30 (11.7%) NA only, 45 (17.5%) SI only, and 83 (32.3%) having low scores on both traits. Type D personality was not significantly related to age, sex, Body Mass Index (BMI), PCI, hypertension, diabetes and smoking. Patients with type D personality displayed significantly higher scores of anxiety and depressive symptoms than non-type D patients did (Table 1). The prevalence rates of anxiety and depression in type D and non-type D patients are shown in Fig. 2. Chinese AMI patients with a type D personality were at increased risk of anxiety (χ2 = 30.095, P < .001) and depression (χ2 = 27.082, P < .001). Patients with type D personality also displayed significantly higher level of lipoprotein(a) (Lp[a]) (P = .048).

**Table 1:** Baseline characteristics and medical treatment

		Non-Type D n = 203(79%)	Type D n = 54(21%)	p-Value
**Clinical characteristics**				
Age (years; mean ± SD)		64.9 ± 13.6	62.0 ± 13.8	0.410
Male sex		151(74%)	42(78%)	0.877
BMI(Kg/m2)		25.13 ± 3.66	24.56 ± 3.98	0.741
Killip (classification)		1.50 ± 0.90	1.69 ± 0.97	0.280
Ejection fraction(%)		50.7 ± 8.9	48.9 ± 8.9	0.430
Prior ACS or revascularization (%)		29/203 (14.3)	11/54 (20.4)	0.273
**Risk factors**				
Active smoking		100(49.3%)	26(48.1%)	0.989
Hypertension		130(64.0%)	36(66.7%)	0.938
Diabetes		85(41.9%)	18(33.3%)	0.523
**AMI & angio characteristics**				
STEMI		137(67.5%)	36(66.7%)	0.993
Anterior MI (STEMI)		62/137 (45.3%)	22/36(61.1%)	0.090
Primary PCI		119/137(86.9)	32/36(88.9)	0.745
Elective PCI (STEMI)		13/137 (9.5%)	3/36 (8.3%)	0.831
No. of diseased vessels		1.70 ± 0.46	1.75 ± 0.44	0.792
IABP		11/203 (5.4%)	3/54 (5.6%)	0.999
**Lab test**				
Hb (g/L)		133.7 ± 24.0	138.1 ± 19.0	0.467
Cr (µmo/L)		104.5 ± 8.47	115.7 ± 20.1	0.848
Peak Cr(µmo/L)		139.1 ± 12.2	148.9 ± 25.3	0.936
BNP/NT-proBNP baseline	Normal	65/203(32.0%)	20/54(37.0%)	0.486
	Mild	69/203(34.0%)	12/54(22.2)	0.098
	Moderate	22/203 (10.8%)	9/54 (16.6%)	0.242
	Sereve	47/203(23.2%)	13/54(24.1%)	0.887
BNP /NT-proBNP Peak	Normal	15/203(7.4%)	4/54(7.4%)	0.996
	Mild	55/203(27.1%)	15/54(27.8%)	0.920
	Moderate	53/203(26.1%)	13/54(24.1%)	0.761
	Severe	80/203(39.4%)	22/54 (40.7%)	0.859
Peak TnI (ng/ml)		8.7 ± 9.2	11.0 ± 13.6	0.341
LDL-C (mmo/L)		3.09 ± 1.11	3.08 ± 1.15	0.998
Lp(a) (mg/L)		118.1 ± 255.7	177.2 ± 200.7	0.048
HbA1c (%)		6.7 ± 1.6	6.3 ± 1.4	0.289
HCY (µmol/L)		16.8 ± 12.4	16.6 ± 16.4	0.992
HADS-A		4.2 ± 3.1	7.4 ± 3.1	< 0.001
HADS-D		4.0 ± 3.4	7.2 ± 3.8	< 0.001

BMI: body mass index; ACS: acute coronary syndrome; STEMI: ST-segment elevation myocardial infarction; PCI: percutanous coronary intervention; IABP: intra-aortic balloon pump; Hb: haemoglobin; Cr: creatine; BNP: B-Type Natriuretic Peptide; NT-proBNP: N-terminal proBNP; TnI: Troponin I; LDL-C: low-density lipoprotein cholesterol; Lp(a):lipoprotein(a); HbA1c: hemoglobin A1c; HCY: homocysteine; HADS-A: Hospital Anxiety and Depression Scale-Anxiety; HADS-D: Hospital Anxiety and Depression Scale-Depression.

#### BNP/NT-proBNP level

Normal (BNP < 100 pg/ml or NT-proBNP < 400 ng/ml); Mild(100–299 pg/ml or 400–1500 ng/ml); Moderate (300–500 pg/ml or 1500–3000 ng/ml); Severe(> 500 pg/ml />3000 ng/ml).

### In-hospital events

There was no difference in length of stay during hospitalization between type D and non-type D patients (10 ± 7days vs. 10.0 ± 7days, p = .759). The incidence of in-hospital cardiac events was higher in type D than in non-type D patients (24.1% vs. 11.3%, χ2 = 5.751, P = .026). See Table 2 for details of in-hospital MACEs. The results of the logistic regression were presented in Table 3. Multivariable logistic regression showed three significant independent predictors of in-hospital MACEs: age (OR = 1.055; 95%CI1.016-1.095, p = .004), type-D personality (OR 3.332; 95% CI 1.149–9.661, p = .014) and killip class(OR 2.275, 95% CI 1.506–3.437, p < .001).

We also established three other models: including only HADS-A, including only HADS-D, and excluding HADS respectively. Indicators with predictive value for events during hospitalization include age, killip class at admission, and type-D personality. The OR values of type-D personality were 3.018 (95% CI 1.016–8.230, p = .031), 3.506 (95% CI 1.269–9.686, p = .016)and 3.052 (95% CI 1.249–7.458, p = .014), which hinted that adding HADS having no significant impact on type-D personality.

**Table 2:** Cardiac Events during hospitalization and their percentages

Events during hospitalization	Non-Type D	Type D	p
acute pulmonary edema	14	9	0.025
cardiogenic shock	6	2	0.778
acute myocardial ischemia /reinfarction	2	1	0.598
ventricular tachycardia /fibrillation	1	0	0.605
Death	0	1	0.052
MACE (%)	23/203(11.3%)	13/54(24.1%)	0.026

**Table 3:** Logistic regression analysis for predictors of in-hospital events

Predictor	Odds ratio	Wald	95% CI	p value
Type D	3.332	4.910	1.149–9.661	0.014
Killip classification	2.275	15.258	1.506–3.437	< 0.001
Age	1.055	7.728	1.016–1.095	0.004
Prior myocardial infarction	1.612	0.638	0.499–5.205	0.425
Ejection fraction	0.976	1.035	0.933–1.022	0.309
LDL-C	1.031	0.022	0.691–1.538	0.881
fasting blood glucose	1.076	1.905	0.970–1.194	0.167
HADS-A	1.025	0.101	0.880–1.195	0.750
HADS-D	0.939	0.809	0.818–1.077	0.368

LDL-C: low-density lipoprotein cholesterol;HADS-A: Hospital Anxiety and Depression Scale-Anxiety; HADS-D; Hospital Anxiety and Depression Scale-Depression.

### Post-discharge endpoints

**Fig. 3:**
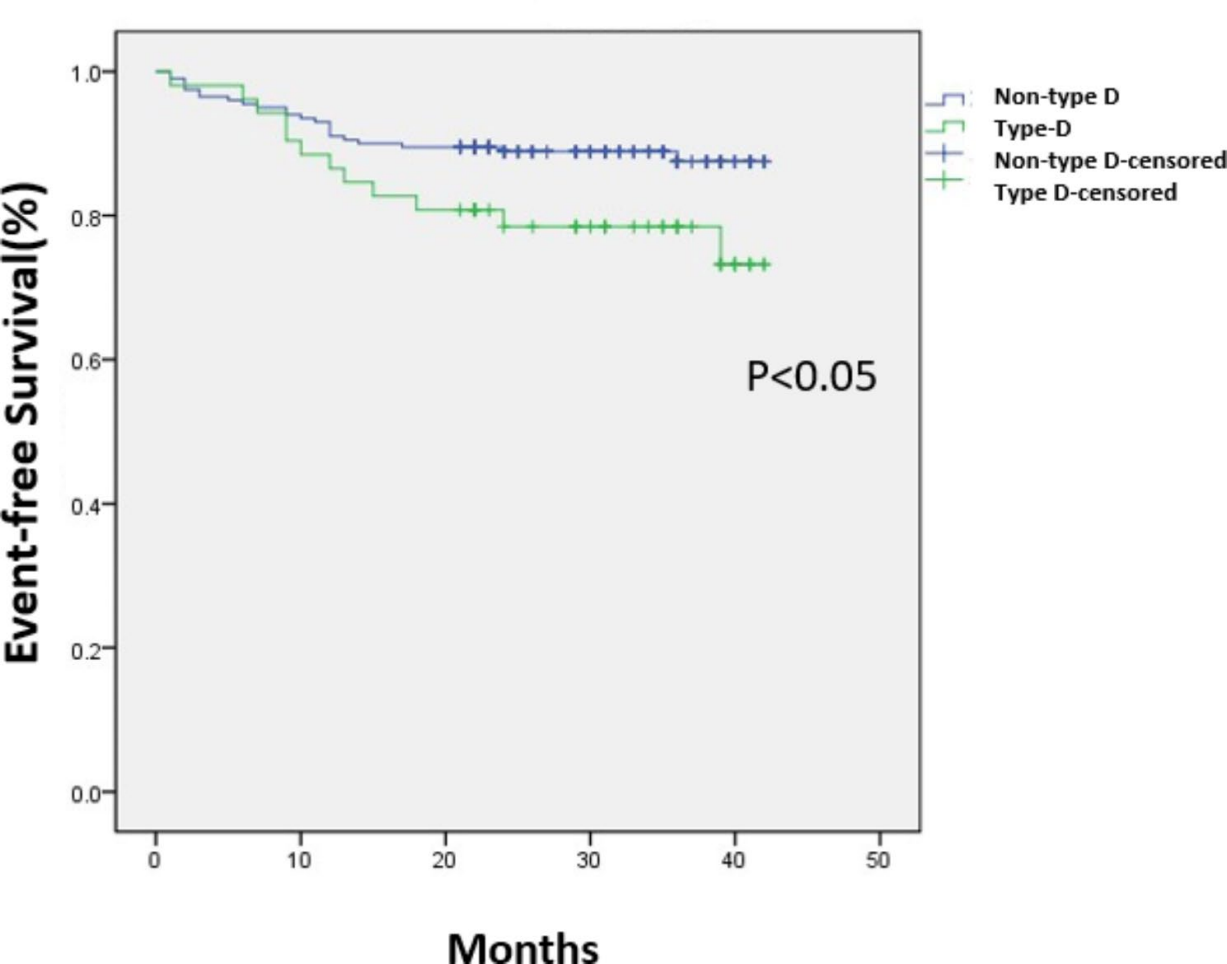
Curves of freedom from the post-discharge MACE in patients with type D and without type D (Kaplan–Meier), p = .043 between groups type D and without type D

The average follow-up time was 31 (23-37.5) months. 4 patients were lost to follow-up with 3 patients in non-type D and 1 in type D group. Three patients died all in non-type D group. One died of severe pneumonia and heart failure. The other two died of Non-ST elevation MI and cardiogenic shock. There were 35 cardiac events in total patients, of which 12 events in type D group and 23 in non-type D group. See details in Table 4, type D patients had higher incidences of cardiac events (23.1% vs. 11.5%, p = .032). The results of the Cox regression were presented in Table 5. χ2 of the Cox regression equation was 16.795 (p = .032). Smoking (HR 2.602; 95% CI1.266–5.347, p = .009) and type-D personality (HR 2.265; 95%CI 1.028–4.988, p = .042) were independent predictors of long-term cardiac events. Kaplan–Meier curves of freedom from the post-discharge events in type D and non-type D group were shown in Fig. 3 (p = .043).

**Table 4:** Major adverse cardiac events during 3-year follow-up

	Non-Type D (n = 200)	Type D (n = 52)	*P value*
MACE%	11.5%(23/200)	23.1%(12/52)	0.032
Unstable angina /revascularization (PCI/CABG)	16	6	0.421
reinfarction	4	6	0.002
cardiac death	3	0	0.374

**Table 5:** Cox regression analysis for predictors of post-discharge MACE

Predictor	Hazard Ratio(HR)	Wald	95% CI	p value
Type D personality	2.265	4.117	1.028–4.988	0.042
Active smoking	2.602	6.768	1.266–5.347	0.009
Hypertension	0.553	1.824	0.234–1.307	0.177
Diabetes	0.552	2.330	0.258–1.184	0.127
No. of diseased vessels	1.405	0.462	0.527–3.740	0.497
Ejection fraction	1.006	0.072	0.965–1.048	0.788
Prior myocardial infarction	1.092	0.027	0.382–3.126	0.869
LDL-C	1.130	1.073	0.897–1.424	0.300

LDL-C: low-density lipoprotein cholesterol.

### Sensitivity analysis

The logistic regression analysis for in-hospital events was also performed by multiplying NA and SI to represent type-D personality. Predictive indicators that were statistically significant for events during hospitalization included age and killip class at admission. The product of NA and SI representing type-D personality also showed a trend towards predicting prognosis, but it did not reached statistical significance. The OR value of the product of NA and SI was 1.005 (95% CI 1.000-1.010, p = .059) and the Wald value was 3.574 when the regression model including HADS-A and HADS-D. While excluding the HADS, the OR value of the product of NA and SI was 1.004 (95% CI 1.000-1.008, p = .051), and the Wald value was 3.793. The results showed consistence with those using type-D dichotomous variables, but the predictive value using product of NA and SI may be lower (See supplemental Table 1). Attempts were also made to use the sum score of NA and SI representing type D personality, NA or SI as separate continuous variables respectively, but they were not found to be independent risk factors for in-hospital events and post-discharge endpoints .

## Discussion

Our study aimed to investigate the association between type D personality and adverse cardiac events in chinese patients after AMI. Over 60% of our selected AMI population were ST-segment elevation myocardial infarction (STEMI) and over 80% of the STEMI patients received primary PCI. Type D personality had a prevalence of 21% in our AMI patients, which is similar to those reported previously [17, 18]. There was a higher prevalence of both anxiety and depression in our type D patients, which supports the viewpoint that type D personality increases the psychological risk factors in AMI patients. The average score of anxiety and depression exceeded seven in our type D group and a cut-off value of seven or higher can combine sensitivity and specificity best when screening for major anxiety and depression [19]. Psychological factors have been implicated in the onset and progression of cardiovascular disease. Anxiety and depression are the most common psychological manifestations after AMI, which are associated with short- and long-term cardiac events after AMI already found by previous studies [14, 15]. Our results showed that the incidence of in-hospital MACEs after AMI in type D is more than twice of that in non-type D group. In addition to age and killip class, type-D personality is the independent predictor for in-hospital MACEs after AMI by multivariable logistic regression analysis. Type D patients also had higher incidences of post-discharge cardiac events by Cox regression analysis. Type-D personality together with smoking are the independent predictors of post-dicharge events in AMI patients, which indirectly confirmed the previous study that type D smokers had a higher incidence of cardiovascular events during the long-term follow-up of AMI [6].

Events definition and endpoints selection are very important in evaluating the prognostic effect of a risk factor in clinical trials. Previous study discussed the heterogeneity in the predictive value of type D personality for cardiac events and mortality [11]. Some type D studies focused on non-cardiac events and reported negative findings, while positive studies selected cardiac endpoints [11]. Type D personality may be more related to cardiac events. So in our study, we selected unstable angina, reinfarction, cardiac revascularization (PCI/CABG) and cardiac death as post-discharge endpoints for cardiac prognosis.

A systematic review compared two popular methods to assess a Type D personality effect using continuous and dichotomous methods, which concluded the dichotomous method may be false positives, with only NA or SI driving the outcome [12]. In our study, the logistic regression analysis for in-hospital events was also performed by multiplying NA and SI to represent type-D personality. Predictive indicators that were statistically significant for events during hospitalization included age and killip class at admission. The product of NA and SI representing type-D personality also showed a trend towards predicting prognosis, but it did not reached statistical significance. The OR value of the product of NA and SI was 1.005 (95% CI 1.000-1.010, p = .059) and the Wald value was 3.574 when the regression model including HADS-A and HADS-D. While excluding the HADS, the OR value of the product of NA and SI was 1.004 (95% CI 1.000-1.008, p = .051), and the Wald value was 3.793. The results showed consistence with those using type-D dichotomous variables, but the predictive value using product of NA and SI may be lower due to the small number of our subjects. And also some critically ill patients who could not cooperate with the DS14 questionnaire and might have a high probability of cardiovascular events were not included in our study. Attempts were also made to use the sum score of NA and SI representing type D personality, NA or SI as separate continuous variables respectively, but they were not found to be independent risk factors for in-hospital events and post-discharge endpoints .

Reza et al. have reported that type D personality is associated with hyperlipidemia in patients with myocardial infarction [5]. In our results, type D personality displayed significantly higher level of blood lipoprotein(a)[Lp(a)], while no difference was shown in low-density lipoprotein cholesterol (LDL-C) level. Lp(a) is an independent risk factor for CHD [20, 21] and more strongly associated with cardiovascular mortality than LDL-C [22].

## Conclusions

In summary, type D personality is an independent predictor of in-hospital and post-discharge cardiac events after AMI in Chinese patients. Adding psychotherapy on optimal cardiological care after AMI may bring benefits on the prognosis of AMI.

### Electronic supplementary material

Below is the link to the electronic supplementary material.

Supplementary Material 1

## Data Availability

The original data will be shared on reasonable request by contacting the corresponding author.

## List of abbreviations

AMI: acute myocardial infarction
CCU: cardiac care unit
DS14: 14-item Type D Scale-14
HADS: Hospital Anxiety and Depression Scale
MACEs: major adverse cardiac events
NA: negative affectivity
SI: social inhibition
OR: odds ratio
CHD: coronary heart disease
PCI: percutanous coronary intervention
HADS-A: Hospital Anxiety and Depression Scale-Anxiety
HADS-D: Hospital Anxiety and Depression Scale- Depression
MI: myocardial infarction
CABG: coronary artery bypass grafting
IQR: interquartile range
BMI: Body Mass Index
ACS: acute coronary syndrome
STEMI: ST-segment elevation myocardial infarction
IABP: intra-aortic balloon pump
Hb: haemoglobin
Cr: creatine
BNP: B-Type Natriuretic Peptide
NT-proBNP: N-terminal proBNP
TnI: Troponin I
LDL-C: low-density lipoprotein cholesterol
Lp(a): lipoprotein(a)
HbA1c: hemoglobin A1c
HCY: homocysteine
