# Supplementary material for: Predictive value of type D personality for cardiac events in Chinese patients with acute myocardial infarction
Source: BMC Cardiovasc Disord. 2023 Nov 14;23:556. doi: 10.1186/s12872-023-03598-w (PMC10648328; doi:10.1186/s12872-023-03598-w)
Supplement: Supplementary file 1 — Supplementary Material 1 [file 12872_2023_3598_MOESM1_ESM.docx]

**Supplemental** **Table 1**

**Logistic regression analysis for predictors of in-hospital events using product of NA and SI representing type-D personality**

**Model 1: including HADS-A and HADS-D**

| **Predictor** | **Odds ratio** | **Wald** | **Standard error** | **95% CI** | **p value** |
| --- | --- | --- | --- | --- | --- |
| Age | 1.051 | 6.940 | 0.019 | 1.013-1.090 | .008 |
| Killip class | 2.347 | 16.320 | 0.211 | 1.552-3.551 | <.001 |
| fasting blood glucose | 1.055 | 0.917 | 0.056 | 0.945-1.177 | .338 |
| HADS-A | 1.012 | 0.022 | 0.081 | 0.864-1.186 | .883 |
| HADS-D | 0.926 | 1.102 | 0.073 | 0.802-1.069 | .294 |
| LDL-C | 1.012 | 0.003 | 0.206 | 0.675-1.516 | .955 |
| Ejection fraction | 0.979 | 0.862 | 0.023 | 0.936-1.024 | .353 |
| Prior myocardial infarction | 1.902 | 1.173 | 0.594 | 0.594-6.090 | .279 |
| NA*SI | 1.005 | 3.574 | 0.003 | 0.880-1.195 | .059 |

**Model 2: without HADS-A and HADS-D**

| **Predictor** | **Odds ratio** | **Wald** | **Standard error** | **95% CI** | **p value** |
| --- | --- | --- | --- | --- | --- |
| Age | 1.051 | 7.377 | 0.018 | 1.014-1.089 | .007 |
| Killip class | 2.492 | 23.995 | 0.186 | 1.729-3.591 | <.001 |
| fasting blood glucose | 1.057 | 1.041 | 0.054 | 0.951-1.174 | .308 |
| LDL-C | 1.021 | 0.011 | 0.198 | 0.693-1.505 | .916 |
| NA*SI | 1.004 | 3.793 | 0.002 | 1.000-1.008 | .051 |

LDL-C：low-density lipoprotein cholesterol，HADS-A：Hospital Anxiety and Depression Scale-Anxiety；HADS-D：Hospital Anxiety and Depression Scale-Depression.

|  |
| --- |
